# Individualized circulating tumor DNA monitoring in head and neck squamous cell carcinoma

**DOI:** 10.1002/cam4.4726

**Published:** 2022-03-30

**Authors:** Ryunosuke Kogo, Tomomi Manako, Takeshi Iwaya, Satoshi Nishizuka, Hayato Hiraki, Yasushi Sasaki, Masashi Idogawa, Takashi Tokino, Ayaka Koide, Noritaka Komune, Ryuji Yasumatsu, Takashi Nakagawa

**Affiliations:** ^1^ Department of Otorhinolaryngology, Graduate School of Medical Sciences Kyushu University Fukuoka Japan; ^2^ Department of Surgery Iwate Medical University School of Medicine Yahaba Iwate Japan; ^3^ Division of Biomedical Research and Development Iwate Medical University Institute of Biomedical Sciences Yahaba Iwate Japan; ^4^ Division of Biology, Department of Liberal Arts and Sciences, Center for Medical Education Sapporo Medical University Sapporo Japan; ^5^ Department of Medical Genome Sciences, Research Institute for Frontier Medicine Sapporo Medical University School of Medicine Sapporo Japan

**Keywords:** circulating tumor DNA, digital PCR, head and neck squamous cell carcinoma, SCC panel

## Abstract

There is no useful biomarker to evaluate treatment response and early relapse in head and neck squamous cell carcinoma (HNSCC). Circulating tumor DNA (ctDNA) is a promising biomarker for detecting minimal residual diseases and monitoring treatment effect. We investigated whether individualized ctDNA analysis could help monitor treatment response and relapse in HNSCC. Mutation analysis of tumor and peripheral blood mononuclear cell (PBMC) DNAs of 26 patients with HNSCC was performed using a custom squamous cell carcinoma (SCC) panel. The identified individualized mutated genes were defined as ctDNA candidates. We investigated whether frequent ctDNA monitoring via digital PCR (dPCR) is clinically valid for HNSCC patients. *TP53* was the most frequently mutated gene and was detected in 14 of 24 cases (58.2%), wherein two cases were excluded owing to the absence of tumor‐specific mutations in the SCC panel. Six cases were excluded because of undesignable and unusable primer‐probes for dPCR. Longitudinal ctDNA was monitored in a total of 18 cases. In seven cases, ctDNA tested positive again or did not test negative, and all seven cases relapsed after initial curative treatment. In 11 cases, after initial curative treatment, ctDNA remained negative and patients were alive without recurrence. Patients who remained negative for ctDNA during follow‐up after initial curative treatment (*n* = 11) had a significantly better prognosis than those who reverted to ctDNA positivity (*n* = 7; *p* < 0.0001; log‐rank test). Individualized ctDNA monitoring using SCC panel and dPCR might be a novel and promising biomarker for HNSCC.

## Graphical abstract

Individualized ctDNA monitoring reflects clinical course and has a potential to detect MRDs and early relapse. individualized ctDNA monitoring using dPCR might be a novel and promising biomarker for HNSCC.

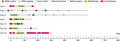

## 1 INTRODUCTION

Head and neck squamous cell carcinoma (HNSCC) is the sixth most common cancer worldwide.^1^, ^2^ Tobacco and alcohol consumption are implicated in the carcinogenesis of HNSCC; likewise, viruses, such as the human papillomavirus (HPV) and Epstein–Barr virus (EBV), are implicated in the carcinogenesis of some HNSCC cases. HNSCC that is HPV‐negative and results from conventional tobacco and alcohol, is characterized by genetic instability and a mutation profile that is quite different from that of HPV‐positive HNSCC.^3^, ^4^ HPV‐negative HNSCC is resistant to chemotherapy and radiotherapy and has a poor prognosis compared to HPV‐positive HNSCC.^5^ The prognosis of patients with recurrent and/or metastatic disease remains poor, wherein the 5‐year survival rate is approximately 50%.^6^ To improve the survival rate of HNSCC, early detection of recurrence and prediction of recurrence risk are clinically important in addition to the development of novel anticancer drugs and therapeutic modalities. However, it is often difficult to detect early relapse using existing diagnostic imaging modalities and serum tumor markers. Moreover, the spatial, epidemiological, genetic, and epigenetic diversity of HNSCC makes predicting recurrence risk even more difficult. Currently, there is no useful biomarker for detecting relapse and evaluating the treatment effect in HNSCC. Existing serum tumor markers, such as SCC antigen and CYFRA (cytokeratin 19 fragment antigen), are not clinically useful because of their low sensitivity and specificity.^7^

Circulating tumor DNA (ctDNA) is a small fragment of tumor‐derived DNA of 150 to 200 base pairs in length and is present in the blood of cancer patients,^8^ which is released from apoptotic and necrotic tumor cells. Due to this tumor‐specific property, liquid biopsy using ctDNA can detect minimal residual diseases (MRDs) and monitor treatment effects.^9^, ^10^ Therefore, the detection of ctDNA has a potential clinical application and is a potential biomarker for HNSCC. Longitudinal surveillance using circulating tumor EBV‐DNA and HPV‐DNA (segment coding for oncoproteins E6/E7) has already been reported.^11^, ^12^, ^13^ However, ctDNA monitoring in most EBV‐ and HPV‐negative HNSCC cases has not yet been established. Because of the properties of EBV‐ and HPV‐negative HNSCC, such as a broad mutation spectrum and the absence of a specific driver gene,^3^ the inability to monitor a single mutated gene makes ctDNA research even more difficult.

We performed next‐generation sequencing (NGS) analysis of tumor and corresponding peripheral blood mononuclear cell (PBMC) DNA and identified tumor‐specific mutated genes, which are ctDNA candidate genes. Subsequently, we designed a case‐specific digital PCR (dPCR) primer and probe to detect ctDNA candidate genes and performed ctDNA monitoring using dPCR.

Here, we indicate that individualized ctDNA monitoring using dPCR accurately reflects the clinical course of HNSCC patients and provides potential clinical applications.

## 2 MATERIALS AND METHODS

### 2.1 Patient and sample collection

All patients in this study provided written informed consent for the IRB‐approved study (Protocol Number: 700–04 and 900–00) at Kyushu University (Fukuoka, Japan) and its related facilities between January 2018 and April 2020. Twenty‐six patients with histologically confirmed HNSCC were enrolled. Tumors were staged according to the 8th edition of the Union for International Cancer Control TNM classification system, except for external auditory canal (EAC) cancer. EAC cancers were staged according to the modified Pittsburgh classification.^14^ Tumor tissues were collected by biopsy before initial treatment or during initial surgery, immediately soaked in Allprotect Tissue Reagent (Qiagen), and then stored at −80°C. Blood samples for cell‐free DNA (cfDNA) were collected using PAXgene Blood ccfDNA Tube (BD Biosciences) and were centrifuged at 1700*g* for 10 min at room temperature within 3 h after blood collection. Plasma (supernatant) was stored at −80°C until the extraction of cfDNA. Blood samples for PBMCs were collected using BD Vacutainer CPT Cell Preparation Tube (BD Biosciences) and were centrifuged at 1700*g* for 20 min at room temperature within 3 h after blood collection. PBMCs were collected from the buffy coat layer and stored at −80°C until DNA extraction.

### 2.2 DNA extraction and panel sequencing

Tumor tissues and corresponding PBMC DNAs were extracted using the QIAamp DNA Mini Kit (Qiagen) according to the manufacturer's protocol. cfDNA series from plasma was extracted using the QIAamp Circulating Nucleic Acid Kit (Qiagen) according to the manufacturer's protocol. Tumor and PBMC DNAs were subjected to amplicon sequencing using the Ion Torrent sequencer (Thermo Fisher Scientific) with a specifically designed SCC panel covering all exons of 31 genes that are frequently mutated in HNSCC, esophageal squamous cell carcinoma, and lung squamous cell carcinoma (Table S1). Approximately 20 ng DNA per sample was used to prepare barcoded libraries with IonXpress barcoded adapters and an Ion AmpliSeq Library Kit Plus (Thermo Fisher Scientific). A total of 20–25 individual barcoded libraries (100 pM each) were pooled and clonally amplified through emulsion PCR using the Ion PI Hi‐Q Chef kit and Ion Chef (Thermo Fisher Scientific). Finally, sequencing was performed on the Ion Proton System using an Ion PI Chip Kit v3 according to the manufacturer's protocol (200 bp read length, Thermo Fisher Scientific).

### 2.3 Identification of ctDNA candidates

Somatic mutations, including single‐nucleotide variants, insertions, and deletions were detected using a variant call algorithm in tumor‐ and matched‐PBMC samples from the Ion Reporter software 5.0 tumor‐normal workflow (Thermo Fisher Scientific) as previously described.^15^ We defined tumor‐specific mutated genes per patient with variant allele frequency (VAF) >10% and total coverage >100 as ctDNA candidate genes. We also selected the gene with the highest VAF among the ctDNA candidates and then designed and generated a primer‐probe for tumor‐specific genes per case using dPCR.

### 2.4 dPCR assay

Patient‐specific primers and probes labeled for wild type (HEX‐conjugated) and mutant (FAM‐conjugated) alleles were specifically designed using Hypercool Primer & Probe technology (Nihon Gene Research Laboratories) to investigate ctDNA by dPCR. The appropriate size (<60–70 bp) of PCR products was designed using 5‐methyl‐dC and 2‐amino‐dA base modifications to adjust the *Tm* value. dPCR was performed using a QuantStudio 3D Digital PCR System (Thermo Fisher Scientific). Before ctDNA monitoring, the synthesized primer and probe sets for each mutation were validated using the corresponding primary tumor DNA.

### 2.5 Statistical analysis

Overall and relapse‐free survival curves were plotted according to the Kaplan–Meier method, with the log‐rank test applied for comparison. The risk was estimated based on overall survival using Cox proportional hazards model. Statistical significance was set at *p* < 0.05. All analyses were performed using JMP Pro 15 (SAS Institute).

## 3 RESULTS

### 3.1 Patient characteristics and sample collection

Twenty‐six patients were enrolled in this study. The median follow‐up was 563 days (range: 96–1033 days) after the initial curative treatment (Table 1). The median age was 71 years (range: 42–86 years old). Fifteen (58%) patients were male, and 19 (73%) were current or former smokers. These patients had oral cavity cancer (*n* = 5), oropharyngeal cancer (OPC) (*n* = 5), hypopharyngeal cancer (HPC) (*n* = 3), laryngeal cancer (*n* = 7), and EAC cancer (*n* = 6). Four out of five OPC patients were immunohistochemically positive for p16, which indicates HPV‐positive OPC. Seventeen of the 26 patients (65.4%) were categorized at Stage IV, and advanced cancer accounted for most cases.

**TABLE 1:** Patient characteristics

Characteristic	*n* = 26 (%)
Median age, years (range)	71 (42–86)
Median follow‐up, days (range)	563 (96–1033)
Sex	
Male	15 (57.7)
Female	11 (42.3)
Smoking	
Current smoker	11 (42.3)
Former smoker	8 (30.8)
Never smoker	7 (26.9)
T stage	
1	4 (15.4)
2	6 (23.1)
3	4 (15.4)
4	12 (46.1)
N stage	
0	10 (38.5)
1	8 (30.8)
2b	3 (11.5)
2c	4 (15.4)
3b	1 (3.8)
UICC Stage	
I	5 (19.2)
II	1 (3.8)
III	3 (11.5)
IV	17 (65.4)
Primary site	
Oral cavity	5 (19.2)
OPC	5 (19.2)
HPC	3 (11.5)
Larynx	7 (26.9)
EAC	6 (23.1)
p16	
Positive	4 (15.4)
Negative	22 (84.6)
Initial curative treatment	
RT	1 (3.8)
CRT	5 (19.2)
IC→CRT	3 (11.5)
Surgery	9 (34.6)
IC→Surgery	2 (7.7)
Surgery→CRT	3 (11.5)
IC→Surgery→CRT	3 (11.5)
Outcome	
DOD	5 (19.2)
NED	21 (80.8)

Abbreviations: CRT, chemoradiotherapy; DOD, died of disease; EAC, external auditory canal; HPC, hypopharyngeal cancer; NED, no evidence of disease; OPC, oropharyngeal cancer; RT, radiotherapy; UICC, Union for International Cancer Control.

**FIGURE 1:**
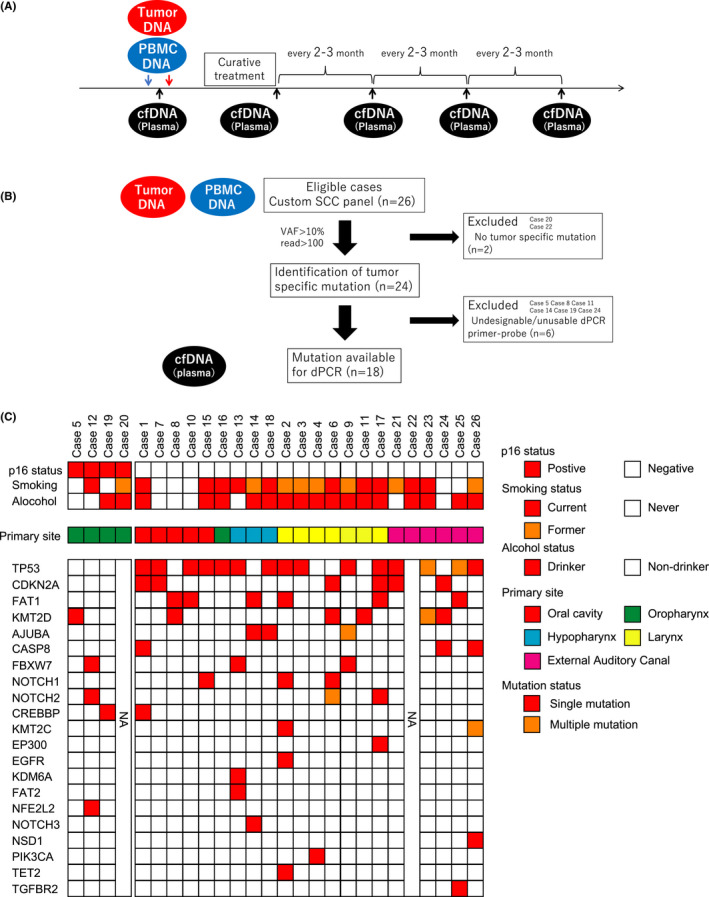
(A) Timing of sample collection. (B) Flow chart of patient eligibility. (C) Tumor‐specific mutation identified from the tumor, and PBMC DNAs using SCC panel. p16 status, smoking, and alcohol status are shown in the top panel. The primary site is shown in the middle panel. Tumor‐specific mutations are shown in the bottom panel. Red boxes show mutated genes with VAF >10% in the primary tumor. The rows are sorted by the gene with the highest mutation frequency. PBMC, peripheral blood mononuclear cell; SCC, squamous cell carcinoma; VAF, variant allele frequency

Figure 1A shows the timing of the sample collection. Tumor and PBMC DNAs were collected before the start of treatment (baseline), and cfDNA (plasma) was collected serially at the baseline, at the end of initial curative treatment, and every 2–3 months during posttreatment follow‐up. Five of the 26 patients died of primary disease during the observation period (Table 1 and Table S2).

### 3.2 Mutation spectrum of HNSCC identified by custom SCC panel

Sequence data were obtained from 26 primary tumors and corresponding PBMCs using a custom SCC panel covering 31 genes that are frequently mutated in SCC (Table S1). The tumor‐specific mutated genes were then identified using Ion, HISAT2, and the BWA algorithm. Among the mutated genes common to these three algorithms, genes with VAFs >10% and reads >100 were defined as ctDNA candidate genes. The SCC panel could not analyze two samples (Cases 20 and 22) due to the lack of identification of tumor‐specific mutations; only the remaining 24 samples were analyzed (Figure 1B).

A summary of the mutation profiles and clinical information is shown in Figure 1C. *TP53* was the most frequently mutated gene and was detected in 14 of 24 cases (58.3%). Consistent with previous reports,^3^, ^4^ mutated *TP53* was detected in 15 of 21 cases (71.4%) in p16‐negative HNSCC, which indicates HPV negativity. In contrast, mutated *TP53* was not detected in p16‐positive HNSCC, which indicates HPV positivity. The number of frequently mutated genes were averagely 1.0 and 2.7 in p16‐positive and ‐negative HNSCC, respectively.

### 3.3 ctDNA detection in pretreatment plasma

We tried to detect tumor‐specific mutated genes (ctDNA candidates) obtained from SCC panel analysis in plasma. We selected the gene with the highest VAF in the ctDNA candidates and then designed and generated a primer‐probe for tumor‐specific genes per case using dPCR (Table S3). Six cases were excluded from ctDNA analysis because of undesignable and unusable primer‐probes for dPCR (Cases 5, 8, 11, 14, 19, and 24, Figure 1B and Table S3). Except for the T1 case (Cases 6 and 10) and T3 EAC cancer (Case 21) case, ctDNA was detectable at baseline. The median of ctDNA VAFs at baseline was 0.4% (range, 0.17–7.4%) (Table S3), and there was no correlation between ctDNA VAFs at baseline and T status/clinical stage/survival (data not shown).

### 3.4 Utility of ctDNA monitoring

**FIGURE 2:**
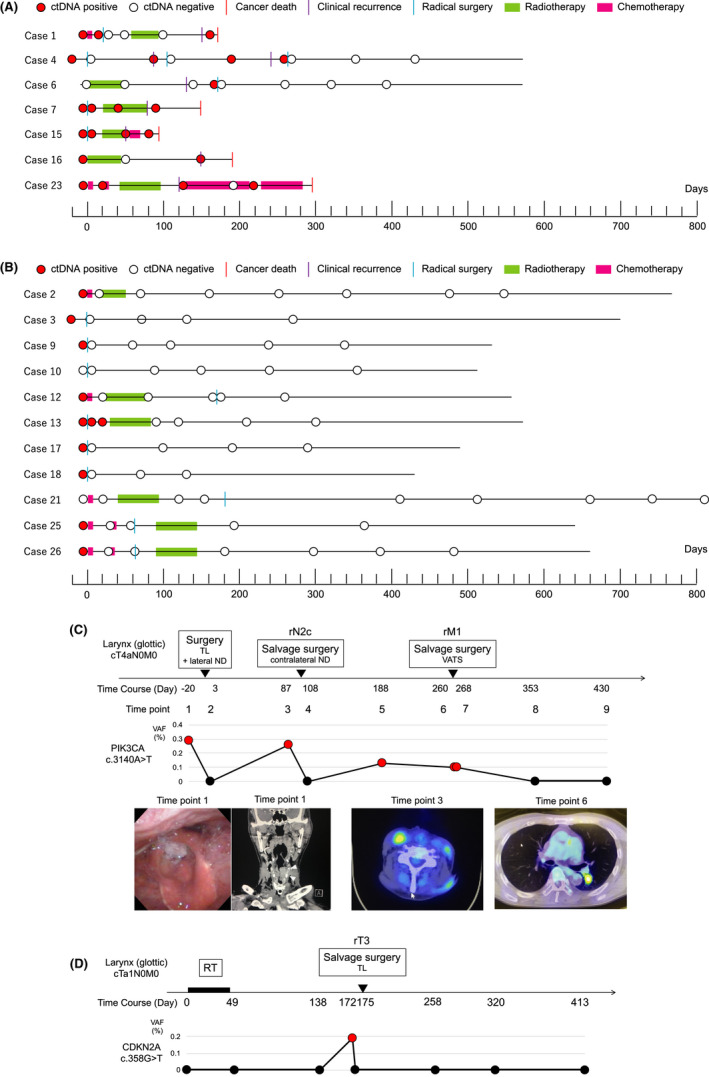
(A) ctDNA monitoring in ctDNA‐positive cases after the initial curative treatment (*n* = 7). ctDNA status and clinical course are represented on horizontal lines. The x‐axis shows the time series of cases. Day 0 indicates the start date of the initial curative treatment. Red and white circles indicate ctDNA‐positive and ‐negative cases, respectively. (B) ctDNA monitoring in ctDNA‐negative cases after initial curative treatment (*n* = 11). ctDNA was never detected in the observation period in two cases (Cases 10 and 21). (C) Case 4, representative case, which is laryngeal cancer (glottic) cT4aN0M0. Clinical course and ctDNA monitoring (*PIK3CA* c.3140A>T) are shown. (D) Case 6, which is laryngeal cancer (glottic) cT1aN0M0. Clinical course and ctDNA monitoring (*CDKN2A* c.358G>T) are shown. TL, total laryngectomy; ND, neck dissection; VATS, video‐assisted thoracic surgery; RT, radiotherapy

The clinical course and longitudinal ctDNA levels are shown in swimmer plots (Figure 2A,B). In 18 cases, ctDNA monitoring using individualized mutant genes was possible longitudinally. However, in two cases (Cases 10 and 21), ctDNA was not detected during the observation period. In seven cases, the ctDNA reverted to positive or did not become negative after the initial curative treatment (Figure 2A). All seven patients had a clinical recurrence or residual disease; wherein five died of the primary disease. In 11 cases, after curative treatment, ctDNA remained negative and the patients were alive without recurrence (Figure 2B).

Representative cases of ctDNA positivity after the initial curative treatment are shown in Figure 2C,D. Case 4, who had progressive laryngeal cancer, received total laryngectomy as an initial curative treatment, followed by contralateral neck lymph node recurrence and salvage surgery with neck dissection (Figure 2C). The ctDNA (*PIK3CA* c.3140A>T) was negative after the first surgery but positive before the neck lymph node recurrence. After the neck dissection, the ctDNA became negative. Moreover, the patient developed a single lung metastasis and underwent salvage surgery with pneumonectomy. Two months before the imaging recurrence by chest CT, the ctDNA became positive. Postoperative ctDNA remained negative, and the patient survived without recurrence. Case 6, who had early laryngeal cancer (glottic cancer) T1aN0M0, was treated with radiotherapy and resulted in CR (Figure 2D). Although ctDNA (*CDKN2A* c.358G>T) was undetectable at baseline, it was positive at the time of local recurrence (rT3). After total laryngectomy, ctDNA remained negative, and the patient survived without recurrence.

### 3.5 Prognostic significance according to ctDNA level posttreatment

**FIGURE 3:**
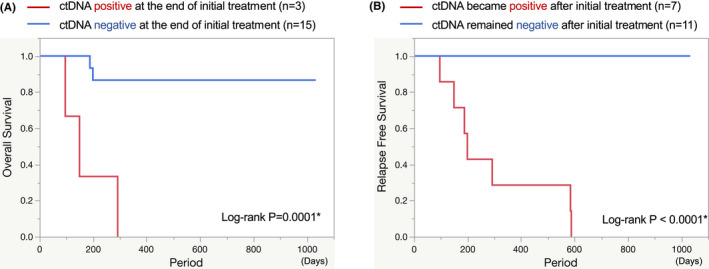
(A) Kaplan–Meier curves showing overall survival according to ctDNA status at the end of initial curative treatment. The end of initial curative treatment means the time of the first plasma collection after the initial curative treatment. P‐value was derived from the log‐rank test. (B) Kaplan–Meier curves showing relapse‐free survival according to ctDNA status during follow‐up after initial curative treatment. *p*‐value was derived from the log‐rank test

Finally, we analyzed survival according to ctDNA status. We investigated whether the ctDNA status in the first plasma sample after the initial curative treatment could predict the prognosis. Patients positive for ctDNA at the end of the initial curative treatment (n = 3) had a significantly worse prognosis than those negative for ctDNA (*n* = 15; *p* = 0.0001; log‐rank test) (Figure 3A). The univariate analysis showed that ctDNA level at the end of the initial treatment was potentially the most critical prognostic factor for survival (*p* = 0.0059, Cox proportional hazards model) (Table S4). In addition, we examined cases of ctDNA positivity and clinical recurrence during follow‐up after the initial curative treatment. Patients who remained negative for ctDNA during follow‐up after the initial curative treatment (*n* = 11) had a significantly better prognosis than those who tested positive for ctDNA after treatment (*n* = 7), and these patients survived without relapse (*p* < 0.0001; log‐rank test) (Figure 3B).

## 4 DISCUSSION

We clarified the clinical validity of nonviral ctDNA monitoring using individualized dPCR primer‐probes in HNSCC patients. ctDNA monitoring in HNSCC has been previously reported in nasopharyngeal carcinoma, where detection of EBV‐DNA in plasma is associated with recurrence rate, overall survival, and relapse‐free survival.^16^, ^17^ Recently, there have been many reports of HPV‐related OPC in which the E6/E7 genes were detected in the plasma, and treatment efficacy and recurrence were monitored.^13^, ^18^ Thus, in some HNSCCs caused by viruses such as HPV and EBV, detecting virus‐derived genes in plasma is expected to be a useful biomarker. However, many other cases of HNSCC progress more rapidly and have a poorer prognosis than virus‐associated HNSCC, and no useful biomarker using ctDNA has been reported.

Moreover, we performed individualized ctDNA monitoring using dPCR. Recently, most ctDNA studies have been performed using NGS‐based methods. Galot et al. reported that ctDNA was detected in 51% of HNSCC patients with local recurrence and/or distant metastasis by a NGS‐based method.^19^ Our study showed that ctDNA was detected in all patients with local recurrence and distant metastasis. Our dPCR‐based individualized ctDNA monitoring could detect ctDNA with higher sensitivity than that detected using the NGS‐based method. Given the minimal amount of blood, rapid turnaround and overall cost, dPCR‐based ctDNA monitoring is a useful application. Particularly due to the rapid turnaround time, dPCR can detect ctDNA in a few hours, and total analysis can be performed within 1 day, including extraction of cfDNA from plasma. The rapid turnaround time allows for early diagnosis and early therapeutic intervention, which may apply to daily clinical practice.

Further, we showed that individualized ctDNA monitoring has a potential to detect MRDs. We often experience difficulties in the detection of MRDs after chemoradiotherapy. Our ctDNA analysis indicated that ctDNA status at the end of initial curative treatment contributed to the prognosis. In the future, it may be necessary to consider therapeutic interventions to improve prognosis, such as adjuvant chemotherapy for patients positive for ctDNA at the end of the initial curative treatment. On the contrary, if the ctDNA remains negative during follow‐up, it may be possible to reduce the number of imaging studies, such as CT and MRI.

Finally, we showed that our ctDNA monitoring may be useful for early relapse detection, as shown in Figure 2C. Even with the clinical use of imaging modalities, we often experience difficulties in the early detection of local recurrence after reconstructive surgery and small lung metastases. In addition, with the developments in chemotherapy, molecularly targeted drugs, and immunotherapy, early diagnosis of recurrence enables early therapeutic intervention and contributes to a better prognosis. ctDNA is a promising biomarker, wherein ctDNA monitoring has been useful in judging early diagnosis of recurrence in colorectal cancer, lung cancer, and esophageal squamous cell carcinoma etc.^20^, ^21^, ^22^ In addition to these cancers, ctDNA monitoring accurately reflected recurrence in HNSCC.

Our data suggest that individualized ctDNA monitoring is an extremely helpful tool for evaluating treatment response and detection of recurrence. However, this study had several limitations. First, our method was not useful for all cases of HNSCC; the detection rate of mutated genes was low in HPV‐related OPC cases, and monitoring of HPV‐related genes, E6/E7, as reported previously, may be superior. We also observed six cases in which a customized dPCR primer‐probe to detect tumor‐specific mutations could not be designed or could not be performed; these six cases did not harbor *TP53* mutation. However, *TP53* mutations in serum were detectable by dPCR in all cases. As described above, *TP53* is the most frequently mutated gene in HPV‐negative HNSCC. Therefore, individualized ctDNA monitoring using dPCR may be more useful in HPV‐negative HNSCC. Second, ctDNA could not be detected at baseline in T1 cases: low tumor burden. Previous reports mentioned that patients with early‐stage cancers harbor little mutated genes per milliliter of plasma, making it difficult to detect ctDNA.^23^ However, in case 6 (laryngeal cancer, T1aN0M0), ctDNA was not detectable at baseline but was detectable at the time of locoregional recurrence (rT3) (Figure 2D). Thus, ctDNA monitoring might be useful for surveillance, even if ctDNA cannot be detected at baseline.

In summary, dPCR‐based ctDNA monitoring using patient‐specific mutations can be a promising, daily use biomarker in HNSCC, particularly HPV‐negative and *TP53*‐mutated HNSCC. ctDNA monitoring may facilitate earlier initiation of salvage therapy. However, this study was a single institutional analysis. It is necessary to confirm the clinical usefulness of individualized ctDNA monitoring in a larger cohort and with a longer follow‐up period.

## CONFLICT OF INTEREST

Drs. Iwaya and Hiraki received grant/research support from Quantdetect. Dr. Nishizuka received grant/research support form Iwate prefecture, Thermo Fisher Scientific, and Geninus. Dr. Nishizuka is a stockholder of Quantdetect. Dr. Hiraki receives consulting fee from Quantdetect. The remaining authors disclose no conflict.

## AUTHOR CONTRIBUTIONS

RK designed this study and wrote original draft. RK and TN contributed to funding acquisition. RK, TM, AK, NK, and RY contributed to collecting the clinical data. RK and TM collected clinical samples, extracted DNA, and performed digital PCR analysis. TI, SN, and HH contributed to a part of digital PCR analysis and provided critical suggestion. YS, MI, and TT contributed to SCC panel analysis. All authors read and approved the final version of the manuscript.

## ETHIC STATEMENT

This study was approved by IRB of Kyushu University (Fukuoka, Japan).

## Supporting information


Table S1‐S4

## Data Availability

All data are available upon request from the corresponding author, except for clinical information. The clinical information is not publicly available due to privacy or ethical restrictions.
